# Safety attitudes in hospital emergency departments: a systematic review

**DOI:** 10.1108/IJHCQA-07-2018-0164

**Published:** 2019-12-08

**Authors:** Naif Alzahrani, Russell Jones, Amir Rizwan, Mohamed E. Abdel-Latif

**Affiliations:** 1 Men, Women and Children’s Health, The Medical School, College of Health and Medicine, Australian National University, Canberra, Australia; 2 Emergency Services Research Group and Health Simulation Centre, School of Medical and Health Sciences, Edith Cowan University, Joondalup, Australia; 3 Saudi German Hospitals Group, Riyadh, Saudi Arabia; 4 Department of Neonatology, Centenary Hospital for Women and Children, Canberra Hospital, Garran, Australia

**Keywords:** Patient safety, Quality improvement, Teamwork, Safety climate, Safety attitude

## Abstract

**Purpose:**

The purpose of this paper is to perform and report a systematic review of published research on patient safety attitudes of health staff employed in hospital emergency departments (EDs).

**Design/methodology/approach:**

An electronic search was conducted of PsychINFO, ProQuest, MEDLINE, EMBASE, PubMed and CINAHL databases. The review included all studies that focussed on the safety attitudes of professional hospital staff employed in EDs.

**Findings:**

Overall, the review revealed that the safety attitudes of ED health staff are generally low, especially on teamwork and management support and among nurses when compared to doctors. Conversely, two intervention studies showed the effectiveness of team building interventions on improving the safety attitudes of health staff employed in EDs.

**Research limitations/implications:**

Six studies met the inclusion criteria, however, most of the studies demonstrated low to moderate methodological quality.

**Originality/value:**

Teamwork, communication and management support are central to positive safety attitudes. Teamwork training can improve safety attitudes. Given that EDs are the “front-line” of hospital care and patients within EDs are especially vulnerable to medical errors, future research should focus on the safety attitudes of medical staff employed in EDs and its relationship to medical errors.

## Introduction

The effective delivery of hospital services and patient care is significantly tied to the safety attitudes and practices of hospital staff and management (Reason, 1993). Indeed, issues related to patient health and safety in hospitals throughout the world have resulted in-patient deaths, prolonged hospitalisations, irreversible disabilities and significant financial costs (Reason, 1993; Abdou and Saber, 2011; Alayed *et al.*, 2014; Allen, 2009; Almutairi *et al.*, 2013; Chaboyer *et al.*, 2013; Duthie, 2006; Profit *et al.*, 2012; Rodriguez-Paz and Dorman, 2008). To address these issues, recent research has focussed on the importance of a hospital safety climate to optimise the effective delivery of patient care. According to The Health Foundation (2011), safety climate focuses on staff perceptions about how safety is managed within their organisation in terms of measurable components. These measurable components include management behaviours, safety systems and employee’s safety attitudes (The Health Foundation, 2011).

Measuring safety attitudes among hospital staff has been widely researched and reported in the literature to provide a lens through which to view and improve the patient safety culture in hospitals (Blegen *et al.*, 2005; Bondevik *et al.*, 2014; Carvalho *et al.*, 2015; Sexton *et al.*, 2011; Steyrer *et al.*, 2013; Yaprak and Intepeler, 2015). Indeed, Sexton *et al.* (2006) maintain that attitudes gauged through surveys of the perceptions of frontline workers within hospitals provide a snapshot of hospital safety culture (Sexton *et al.*, 2006). Safety attitudes have been investigated in a range of countries and different hospital departments. For example, Allen (Allen, 2009) employed the Safety Attitudes Questionnaire (SAQ; Sexton *et al.*, 2006) to establish the safety culture in the maternity services of two Australian hospitals. He found the optimal safety culture was lacking across six safety domains, especially in the domain of management support and working conditions. Moreover, the safety culture was influenced by poor communication when the need for care escalated, lack of supervision of junior staff, issues with staffing, skill mix and low morale.

Along with the significant research focus on safety attitudes within hospital settings, there have been several systematic reviews of findings relating to patient safety attitudes. These have included systematic reviews relating to the safety attitudes of hospital staff in Arab countries (Elmontsri *et al.*, 2017) and hospital in-patient settings (Weaver, 2013). Other systematic reviews have investigated research connecting patient safety attitudes and patient outcomes to determine nurse-sensitive patient outcomes in hospital settings (DiCuccio, 2015), studies on patient safety issues and practices in emergency medical services (Bigham *et al.*, 2012) and studies on patient safety culture strategies to improve the hospital patient safety climate (Morello *et al.*, 2013). Yet, there has been no systematic review of the state of research literature on safety attitudes of health staff employed in hospital emergency departments (Eds). This would appear to be an important issue to clarify, given that EDs are the “front-line” of hospital care (Rigobello *et al.*, 2017) and patients within EDs are especially vulnerable to medical errors (Shaw *et al.*, 2009). The primary objective of this study was to perform a systematic review of published research on the patient safety attitudes of health care professional staff employed in hospital EDs.

## Methods

### Data sources and search strategy

To meet the objective of this study, an electronic literature search was conducted in July 2018 using six different science, health and medicine focussed research databases: PsychINFO, ProQuest, MEDLINE, EMBASE, PubMed and the Cumulative Index to Nursing and Allied Health Literature (CINAHL). No limitations were set on the date of publications; however, search filters were used to limit search hits to publications published in English. The database search strategy entailed initial uses of a broad search term to capture a wide body of studies relevant to the review. Thus, the search process included combinations of the terms “Hospital Emergency department staff”, “Patient Safety attitudes”, “Safety Culture”, “Safety Climate”, “Medical Errors” and “Adverse Events” as well as combinations of MeSh terms “Safety Management”, “Patient Care Team” and “Attitude of Health Personnel” (Appendix 1).

### Inclusion criteria and study selection

**Figure 1:**
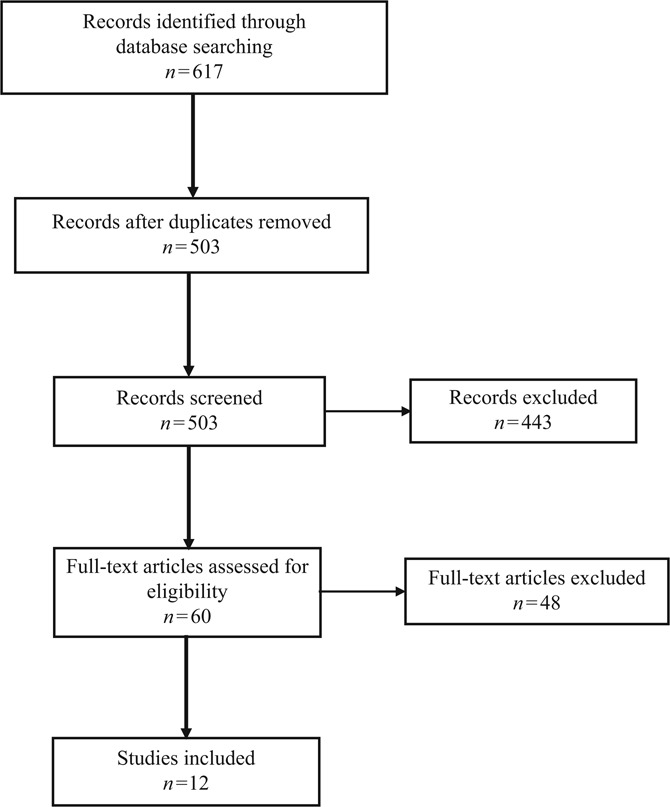
PRISMA flow diagram

Two reviewers performed an assessment of the eligibility of potential studies for inclusion in the review of research on safety attitudes in EDs. All identified records from the aforementioned database searches (total of *n* = 617) were imported into EndNote citation software where duplicates were first identified and then removed. The 503 remaining titles and abstracts were screened against the predetermined inclusion and exclusion criteria such that studies where attitudes of hospital staff towards patient safety had been assessed and/or measured were included at this point of the review. Based on this set of criteria, an additional 443 studies were further excluded from the review, leaving a total of 60 eligible research articles. A final inclusion/exclusion criterion was then applied by removing articles where the study setting did not include a hospital ED. From this investigation, a total of 48 papers were excluded from the final review leaving 12 full-text research papers for in-depth analysis and review. Full-text articles were retrieved from an electronic library and examined in detail for the study design, sample, measures and findings. The study selection process is summarised in Figure 1 using the PRISMA flow diagram (Moher *et al.*, 2009).

### Data extraction and quality appraisal

Data were extracted included study sample and setting, type and number of participants, study design, variables and measurement tools and study findings. The quality of the reviewed articles was assessed through the NIH Quality Assessment Tool for Observational Cohort and Cross-Sectional Studies (NIH, 2017) which gives a score out of 14 to indicate the quality of research studies.

## Results

In total, 12 studies met the inclusion criteria. The methodological characteristics, measures and findings from these articles are summarised in Table I. The studies covered a wide variety of settings including three studies in the USA, two studies in Sweden, and one study each in Australia, Brazil, Cyprus, Denmark, Iran, China and the Netherlands. Four studies were conducted in a single ED site, two studies were conducted in two sites, and six studies included participants from multiple ED sites (from 5 to 62). Whereas ten of the studies were quantitative cross-sectional designs with survey methods, two studies entailed the use of a qualitative phenomenological methodology with semi-structured interviews. Of the ten cross-sectional studies, two used a repeated measures design whereby participants completed a survey prior to and after a safety quality improvement intervention.

### Participants and measures

Across the 12 studies there were a total of 7,645 participants. Most participants were either nurses or physicians working in an ED. In the ten cross-sectional studies, participants completed a validated measure of patient safety culture attitudes, whereas participants in the qualitative studies answered open-ended questions about patient safety attitudes. Three cross-sectional studies also measured the number of adverse patient events to compare against safety attitudes.

## Findings

The two studies that used a quantitative repeated measures design (Burstrom *et al.*, 2014; Lisbon *et al.*, 2016) entailed the use of a team building intervention to test the effects of the intervention on the safety attitudes of participants. Together, the interventions had some success because, post-intervention, the safety culture attitudes demonstrated improved teamwork and communication. Nevertheless, the safety attitudes of physicians and nurses from EDs were generally less than positive in both study settings even after the intervention.

A further eight studies were survey-based using cross-sectional designs where ED staff completed different measures of safety attitudes on one occasion (Rigobello *et al.*, 2017; Shaw *et al.*, 2009; Camargo *et al.*, 2012; Lambrou *et al.*, 2015; Rasmussen *et al.*, 2014; Tourani *et al.*, 2015; Verbeek-Van Nord *et al.*, 2014; Wang *et al.*, 2014). In two of these studies, physicians’ safety attitudes were reported as more positive than nurses (Shaw *et al.*, 2009; Verbeek-Van Nord *et al.*, 2014) although overall safety attitudes reported in six of the eight cross-sectional studies were generally low, especially on teamwork, in-patient coordination and management support. In contrast, job satisfaction was comparatively high in one study (Rigobello *et al.*, 2017). Moreover, the findings from three studies showed more positive safety attitudes were associated with teamwork, communication and management support (Verbeek-Van Nord *et al.*, 2014), improved management of EDs and the presence of an ED safety committee (Shaw *et al.*, 2009), and leadership and autonomy, control over practice, and cultural sensitivity (Lambrou *et al.*, 2015). Importantly, three studies compared safety attitudes to patient adverse event data (Camargo *et al.*, 2012; Rasmussen *et al.*, 2014; Wang *et al.*, 2014) with only one study showing the number of adverse events was related to a poor safety and team climate, poor inter-departmental working relationships, and increased cognitive demands (Tourani *et al.*, 2015). Of the reviewed studies, only two employed a qualitative research design (Grover *et al.*, 2017; Källberg *et al.*, 2017). These studies reported similar findings in that teamwork and team support, workload, and communication and organisational failures were found to be critical to enhanced patient safety.

### Quality rating

The quality rating of each study was assessed through the NIH Quality Assessment Tool for Observational Cohort and Cross-Sectional Studies (NIH, 2017). According to the rating system, the quantitative intervention studies by Burstrom *et al.* (2014) and Lisbon *et al.* (2016) were the highest quality research with a score of 8/14 and 6/14, respectively (Burstrom *et al.*, 2014; Lisbon *et al.*, 2016). The fact that both studies employed an intervention to test the direct effect of an independent variable (IV) on a dependant variable (DV) distinguished the quality of these studies from the other studies in the review. Nevertheless, the study by Lisbon *et al.* (2016) had lower quality because the study population was not clearly defined and over 20 per cent of the participants were lost to follow-up.

The quality rating of the eight other quantitative studies in the review (Rigobello *et al.*, 2017; Shaw *et al.*, 2009) was quite low (between 3/14 and 6/14) and reflected the fact that each study employed a cross-sectional survey design with little control over extraneous or intervening variables where only the relationship between the IV and DV could be established. Similarly, the qualitative studies by Grover *et al.* (2017) and Källberg *et al.* (2017) were rated low (2/14) because each study did not employ a systematic sampling procedure or use valid and reliable measures (Grover *et al.*, 2017; Källberg *et al.*, 2017). Overall, each of the 12 studies failed to justify the sample size through appropriate use of power analysis and estimates of effect size. Furthermore, only one study (Wang *et al.*, 2014) made an adjustment in analysis to take into account key potential confounding variables such as the gender, profession and years of practice of participants.

## Discussion

The primary objective of this study was to perform a systematic review of published research on the patient safety attitudes of health care professional staff employed in hospital EDs. This systematic review of the current literature identified 12 studies, including 10 quantitative studies and 2 qualitative studies that met the inclusion criteria of studies where the safety attitudes of health care professionals from hospital EDs was ascertained. Given the number of studies to have investigated the safety attitudes of the front-line emergency staff of hospitals is comparatively few and patients within hospital EDs are especially vulnerable to medical errors (Shaw *et al.*, 2009), there is justification for addressing the lack of research on the safety attitudes of emergency hospital staff in future studies.

Furthermore, additional research into the safety attitudes of hospital staff is justified because the current systematic review revealed the overall methodological quality of the reviewed studies was comparatively low. Despite some of the reviewed studies having large participant numbers which contribute to the validity of the findings, all the quantitative studies employed cross-sectional research designs which undermines the internal validity of the findings such that it is not possible to observe the direct effects of an IV on a DV. Nevertheless, two higher quality studies employed team building interventions that showed safety culture attitudes improved teamwork and communication post-intervention (Burstrom *et al.*, 2014; Lisbon *et al.*, 2016).

The importance of teamwork and communication to safety attitudes in hospital EDs was also evident in three of the other reviewed quantitative studies. In two of these studies (Shaw *et al.*, 2009; Camargo *et al.*, 2012), more positive safety attitudes were associated with teamwork, communication and management support as well as improved management of EDs and the presence of an ED safety committee. Similarly, one reviewed qualitative research design reported teamwork and team support as critical to enhanced patient safety (Grover *et al.*, 2017). Nevertheless, teamwork and management support are often rated comparatively low on multidimensional safety attitude scales (Chaboyer *et al.*, 2013; Profit *et al.*, 2012; Alzahrani, 2015) such as the studies reviewed here show (Shaw *et al.*, 2009; Verbeek-Van Nord *et al.*, 2014). It would appear from the literature and the review of research reported here that human resource issues like teamwork and management support are related to lower safety attitudes of hospital staff and that interventions to improve these factors in the EDs of hospitals are likely to impact positively on safety attitudes.

The findings from this review that ED physicians’ safety attitudes were reported as more positive than nurses (Shaw *et al.*, 2009; Verbeek-Van Nord *et al.*, 2014) is consistent with previous research in other hospital departments. For example, Thomas *et al.* (2003) reported nurses rated the quality of collaboration and communication with physicians to be lower than the ratings of doctors. As surmised by Thomas, the findings are likely to be associated with differences in status/authority between nurses and physicians, differential responsibilities and training, gender issues, and nursing and physician cultures. Nevertheless, the findings of this review suggest the safety issues associated with the human resource components of a hospital ED are a particular focus for nurses.

Altogether, the findings contribute to the literature by being one of the first studies to systematically review the safety attitudes of health professionals in hospital EDs. Although the numbers of studies on this topic are limited, they do show that teamwork, communication and management support are central to positive safety attitudes, and that teamwork training can improve safety attitudes. Nevertheless, a strength of three of the reviewed studies was an investigation of the relationship between safety attitudes and adverse patient events (Camargo *et al.*, 2012; Rasmussen *et al.*, 2014; Wang *et al.*, 2014) with one study showing the number of adverse events was related to a poor safety and team climate, poor inter-departmental working relationships, and increased cognitive demands (Rasmussen *et al.*, 2014). Yet, the assumed relationship between safety attitudes and hospital error rates has not been clearly and unequivocally shown in the research literature on hospital safety (Steyrer *et al.*, 2013; Ausserhofer *et al.*, 2012). Given that EDs are the “front-line” of hospital care (Rigobello *et al.*, 2017) and ED patients are especially vulnerable to medical errors (Shaw *et al.*, 2009), future research on the safety attitudes of medical staff employed in hospital EDs and how they relate to medical errors is warranted.

## Figures and Tables

**Table I:** Methodological characteristics of reviewed studies

Study	Sample and setting	Design	Variables and measurement tool	Study findings
Burstrom *et al.* (2014)	Participants were physicians and nurses recruited into the study to complete a questionnaire pre- and post-intervention. They were sampled from the emergency department of two hospitals in two different cities in central Sweden: a county hospital and a university hospital. There was relatively equal gender distribution of 92 physicians and 83 nurses among participants at the country hospital and similarly with 35 physicians and 99 nurses (post-intervention participant numbers)	A repeated cross-sectional survey study where participants completed the Hospital Survey on Patient Safety Culture questionnaire before and after a quality improvement project	The 51-item Hospital survey on Patient Safety Culture was administered pre- and post-intervention. The Swedish version of the survey is a reliable and valid measure (Magid *et al.*, 2009) and includes 15 dimensions with one to four items answered on a 5-point Likert scale	The overall rating of safety culture on most dimensions by doctors and nurses at both hospitals and at both measurement points was low (below the midpoint). However, a higher score was measured post-intervention on two dimensions with participants from the country hospital: teamwork within hospital and communication openness. At the university hospital, a higher score was measured at follow-up for the two dimensions: teamwork across hospital units and teamwork within hospital
Camargo *et al.* (2012)	The survey study was conducted in 62 urban EDs across 20 US states. There were 3,562 participants consisting of nurses (52.5%), physicians (22.2%) and other health personnel (25.3%)	A quantitative, descriptive cross-sectional study with survey methods	A 50-item Safety Climate questionnaire with 9-subscales was administered. The scale was reported to be a reliable and valid measure of Safety Climate (Patterson *et al.*, 2010) with each item answered on 5-point Likert scale. Data were also collected on the number of adverse events and near misses in each ED	The overall rating of Safety Climate was 3.5/5 and was especially low on the subscale of Inpatient Coordination (2.4). No data were provided to compare safety climate as a function of profession. A higher safety climate score was not associated with the number of adverse events but was significantly associated with a higher incidence of intercepted near misses
Grover *et al.* (2017)	The study was set in a single major metropolitan emergency department in Melbourne, Australia. Participants were 12 registered nurses (9 female and 3 male)	A qualitative phenomenological study to address the question what are emergency nurses’ perceptions and attitudes towards teamwork?	Five semi-structured interview questions to ascertain and measure attitudes towards the teamwork aspect of patient safety climate	Participants perceived teamwork as an effective construct in resuscitation, simulation training, patient outcomes and staff satisfaction. Team support through back-up behaviour and leadership were perceived as critical elements of team effectiveness. Times were also reported when teamwork failed due including inadequate resources and skill mix
Källberg *et al.* (2017)	The study was conducted in 2 hospital EDs in Sweden at one large urban hospital and one medium-size country hospital. Participants were 10 physicians and 10 registered nurses	A qualitative phenomenological study using semi-structured interviews to investigate patient safety risks	Individual semi-structured interviews with a series of questions to describe events and situations in the ED where patient safety was compromised	Four main categories of patient safety risk were derived from inductive content analysis of the interview data: high workload, lack of control, communication failure and organisational failures
Lambrou *et al.* (2015)	EDs in 5 public general hospitals in Cyprus. Participants 174 nurses and 50 physicians	A descriptive correlational study to measure the association between perceptions of professional practice environment and patient safety	The 39-item Revised Professional Practice Environment (RPPE) Scale measures eight professional practice environment characteristics [Erickson]. A 60-item SAQ adapted for ED environments to measure perceptions of safety culture (Agency for Healthcare Research and Quality, 2016)	Physicians assessed the professional practice environment more positively than nurses. The mean SAQ score was 3.18/5 and safety culture was significantly predicted by three RPPE subscales: Leadership and autonomy, Control over practice, and cultural sensitivity
Lisbon *et al.* (2016)	The study setting was an emergency department of an academic hospital in the USA. Participants were 113 emergency department staff including physicians, nurses and ancillary personnel at time 1 of the study; however, only 59 participants completed the full study	A repeated cross-sectional survey study where participants completed questionnaires on day 1, 45 and 90 of TeamSTEPPS training to develop a high-functioning team to improve patient safety	TeamSTEPPS Knowledge Test (Sarac *et al.*, 2010) is a 21-question multiple-choice format exam to measure patient safety knowledge. The AHRQ hospital survey on patient safety (Sarac *et al.*, 2010) assesses staff attitudes on patient safety culture in the hospital setting	*χ*^2^ tests showed knowledge and attitudes significantly improved 45 days from baseline and were sustained by day 90
Rasmussen *et al.* (2014)	The study setting was an emergency department (ED) at a Danish regional hospital. A total of 98 nurses and 26 doctors were participants in the study	A quantitative, descriptive cross-sectional study with survey methods to measure the relationship between work environment and adverse events (AEs)	The SAQ was used to measure safety climate and teamwork. A Danish scale to measure reporting behaviour and learning environment. Involvement in an adverse event during the preceding month was reported using 43 items covering the classification of AEs from the Danish Patient Safety Database	There were significant positive relationships between the number of reported AEs and poor safety climate, poor team climate, poor inter-departmental working relationships and increased cognitive demands
Rigobello *et al.* (2017)	The study setting was an emergency department of a university teaching hospital in Sao Paulo, Brazil. There were 125 participants recruited into the study which was made up of mostly nurses, physicians and other health professionals	A quantitative, descriptive cross-sectional study with survey methods	The main variable was safety attitudes which were measured with the Portuguese version of the Safety Attitudes Questionnaire (Sexton *et al.*, 2006). The SAQ measures six dimension of safety attitudes: stress recognition, perceptions of management, safety climate, teamwork climate, job satisfaction and working conditions	Of the six dimensions, participants only rated job satisfaction positively. The other dimensions were rated negatively, especially perceptions of management, safety climate and working conditions
Shaw *et al.* (2009)	The setting was 21 emergency departments in paediatric hospitals in the USA. A total of 1,747 staff members (49%) responded to a survey on the climate of safety including nurses, physicians and medical technicians	Quantitative cross-sectional design with survey methods	A validated survey to assess characteristics of emergency department physical structure, staffing patterns, overcrowding, medication administration, teamwork and methods for promoting patient safety (checked as either absent or present). A validated survey on the climate of safety. The survey has 19 questions regarding staff perceptions of the climate of safety each using a 5-point Likert-type scale	There was a wide range (28–82%) in the proportion reporting a positive safety climate across the 21 sites. Physicians’ ratings of the climate of safety were higher than nurses’ ratings. Characteristics associated with an improved climate of safety were a lack of ED overcrowding, a sick call back-up plan for physicians and the presence of an ED safety committee
Tourani *et al.* (2015)	The study setting was 11 EDs in hospitals affiliated with the Tehran Medical Science University in Iran. There were 270 participants comprised of doctors and nurses	Quantitative cross-sectional design with survey methods	The standard questionnaire of Hospital Survey on Patient Safety Culture (HSOPSC) was the main measurement tool which includes 42 statements that focus on 12 different aspects of patient safety	Half of the participants believed there was a problem in the error prevention procedures and systems, 30% reported their supervisor does not pay attention to their recommendations to improve the patients, and 40% reported hospital management show interest in the patients’ safety only when something goes wrong. More than half of the participants believed the nature of tasks in emergency wards, high workload, poor staffing and more than 40 h work a week has caused the staff in the emergency wards to work intensively with 57% of participants reporting there is lack of coordination among the wards
Verbeek-Van Nord *et al.* (2014), Verbeek-Van Nord *et al.* (2014)	The setting was 33 emergency departments in the Netherlands. Participants were 480 nurses, 159 physicians and 91 other health professionals	Quantitative cross-sectional design with survey methods	A Dutch version of the 40-item Hospital Survey on Patient Safety Culture (Alzahrani, 2015) to measure safety culture covering 11 patient safety culture dimensions	Six dimensions of safety culture were positively associated with the reported level of patient safety: teamwork across units, frequency of event reporting, communication openness, feedback about and learning from errors, hospital management support for patient safety. Physicians rated overall perceptions of patient safety higher than nurses
Wang *et al.* (2014)	The study setting was 8 hospitals in Guangzhou, China to include one medical unit, one surgical unit, one intensive care unit and one emergency department from each hospital. A total of 463 registered nurse were participants in the study	Quantitative cross-sectional design with survey methods	The 42-item Hospital Survey on Patient Safety Culture (HSOPSC) measures 12 patient safety culture dimensions. A 7-item adverse events questionnaire was employed to measure the frequency of different adverse patient events from 0 = never to 6 = every day	Data were pooled across the four units and eight hospitals and showed an average patient safety culture score of 3.46/5 with most dimensions being rated below strong, especially hospital management support for patient safety and overall perceptions of safety. A higher mean score on two dimensions of the HSOPSC, “Organizational Learning-Continuous Improvement” and “Frequency of Event Reporting”, was significantly related to lower occurrence of adverse events

## Appendix. PubMed search strategy

“patient safety culture” OR “safety culture survey” OR “safety attitude questionnaire” OR “safety attitudes questionnaire” OR “safety attitude” OR “patient safety practice” OR (“Hospital Survey” AND “patient safety culture”) OR (“Patient Safety Culture” AND “survey”) OR “patient safety climate” OR “attitude of health personnel” OR (“safety culture” OR “safety practice” OR “safety climate”)

AND

(emergency OR “emergency department” OR hospital OR hospitals OR “emergency room” OR attitude OR attitudes OR “adverse events” OR “medical errors” OR “adverse events” OR “hospital staff” OR “safety management” OR patients OR patient OR “primary care”) OR “hospital patient climate safety scale”.
